# American Public Health Problems

**Published:** 1915-10

**Authors:** 


**American Public Health Problems.** Balance of Mortality, 1864-1888, 1889-1913. (Rate per 100,000 of population). New York, Boston, Philadelphia, New Orleans:

| | 1864-1888 | | 1889-1913 | |
|---|---|---|---|---|
| | Deaths | Rate | Deaths | Rate |
| Smallpox | 23,799 | 39.5 | 3,308 | 2.4 |
| Asiatic Cholera | 4,506 | 7.5 | 10 | 0.01 |
| Yellow Fever | 8,469 | 14.0 | 821 | 0.6 |
| Scarlet Fever | 39,983 | 66.3 | 25,560 | 18.8 |
| Diphtheria and croup | 74,274 | 123.2 | 79,396 | 58.3 |
| Typhoid and typhus fevers | 32,042 | 53.1 | 33,573 | 24.7 |
| Pulmonary tuberculosis | 220,048 | 364.9 | 303,862 | 223.3 |
| Pneumonia | 113,712 | 188.5 | 315,648 | 232.0 |
| Stomach and intestinal diseases | 164,598 | 298.6 | 266,991 | 196.2 |
| Heart diseases | 62,565 | 103.7 | 223,991 | 164.6 |
| Nephritis | 47,479 | 78.7 | 179,258 | 131.7 |
| Cancer | 27,305 | 46.4 | 98,085 | 72.1 |

